# Pro-haloacetate Nanoparticles for Efficient Cancer Therapy *via* Pyruvate Dehydrogenase Kinase Modulation

**DOI:** 10.1038/srep28196

**Published:** 2016-06-21

**Authors:** Santosh K. Misra, Mao Ye, Fatemeh Ostadhossein, Dipanjan Pan

**Affiliations:** 1 Departments of Bioengineering, Materials Science and Engineering and Beckman Institute, University of Illinois at Urbana-Champaign, Mills Breast Cancer Institute, and Carle Foundation Hospital, Urbana, Illinois 61801, USA

## Abstract

Anticancer agents based on haloacetic acids are developed for inhibition of pyruvate dehydrogenase kinase (PDK), an enzyme responsible for reversing the suppression of mitochondria-dependent apoptosis. Through molecular docking studies mono- and dihaloacetates are identified as potent PDK2 binders and matched their efficiency with dichloroacetic acid. *In silico* screening directed their conversion to phospholipid prodrugs, which were subsequently self-assembled to pro-haloacetate nanoparticles. Following a thorough physico-chemical characterization, the functional activity of these novel agents was established in wide ranges of human cancer cell lines *in vitro* and *in vivo* in rodents. Results indicated that the newly explored PDK modulators can act as efficient agent for cancer regression. A Pyruvate dehydrogenase (PDH) assay mechanistically confirmed that these agents trigger their activity through the mitochondria-dependent apoptosis.

Recent studies suggest that directing the cancer-specific metabolic and mitochondrial remodeling may offer high level of selectivity in cancer treatment^1^,^2^,^3^. Pyruvate dehydrogenase kinase (PDK) is a mitochondrial enzyme (gate keeper) activated in wide ranges of cancers and consequences in the selective inhibition of pyruvate dehydrogenase, a complex of enzymes that converts cytosolic pyruvate to mitochondrial acetyl-CoA, the substrate for the Krebs’ cycle to suppress mitochondrial apoptosis in cancer cells^4^,^5^,^6^. Selective inhibition of PDK with either small interfering RNAs (siRNAs)^7^ or small molecule drugs^8^ alters the metabolism of cancer cells from glycolysis to glucose oxidation and reverses the suppression of mitochondria-dependent apoptosis. Very recently dichloroacetic acid (DCA), an orally available small molecule has been shown to inhibit the PDK^9^,^10^,^11^ activities resulting in suppression of tumor (e.g. lung, breast, brain) growth pre-clinically *in vitro* and *in vivo* and also in a recent clinical trial. DCA is an inexpensive generic compound and the fact that it has long been used in humans for over 40 years, provided a strong rationale for its rapid clinical translation. DCA reverses the mitochondrial remodeling, unlocking the cancer cells from a state of apoptosis resistance. Not only does this direct the cancer cell to unrestraint its preferred metabolic process, but it turns on the cell’s “suicide switch” as well. This happens because mitochondria are the primary regulators of apoptosis, or cellular suicide. They are programmed to react to aberrations by pushing the cell’s self-destructive switch. DCA has been shown to directly cause cancer cell apoptosis and works synergistically with other cancer therapies, such as radiation, gene therapy, and viral therapy^12^,^13^,^14^,^15^. However, DCA is not a natural agent and is produced as by product of water chlorination process. It is easily absorbed by body and can permeate through blood brain barrier. Due to a rather easy access to the brain, the molecule can exert all sorts of unexpected and severe neurological effects. So, to control the premature release of free haloacetates in circulatory system, a phospholipid-based prodrug (pro-haloacetate) was designed which can be cleaved to release the haloacetates in a selective manner inside the cancer cell. The prodrug molecules self-assemble in presence of a co-surfactant polyethyleneglycol cetyl ether (PEGCE) to generate stabilized nanoparticles. This approach also precludes the possibility of permeation of haloacetates through blood-brain-barrier (BBB) to impart neurotoxicity. Thus, a pro-haloacetate-NP would be an excellent strategy to control the off-target toxicity and manipulating a cancer cell specific delivery to fully appreciate the potential of this metabolic-modulating agent.

## Results and Discussion

**Figure 1:**
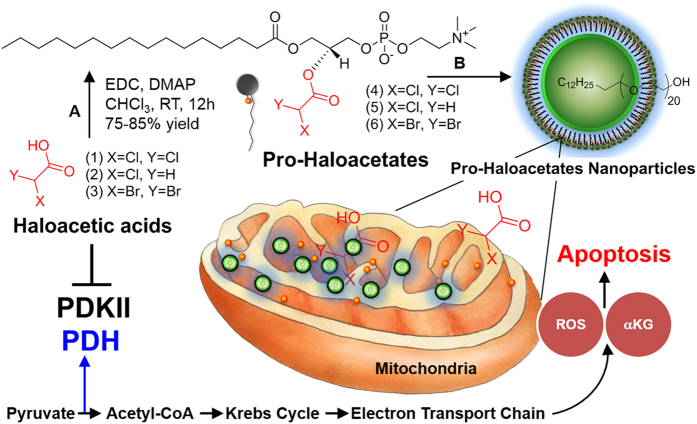
Repairing cancer cell suicide mechanism (programmed cell death). A modulation of PDK by nano-enabled delivery of haloacetates. (**A**) 1-Ethyl-3-(3-dimethylaminopropyl)carbodiimide/DMAP, RT, 12 h, (**B**) polyethylene glycol cetyl ether, self-assembly, H_2_O:THF (4:1, v/v).

Towards improving the anti-cancer potential of haloacetates, we decided, first, to study the binding of a set of seven haloacetic acid molecules to PDK isoforms, and second, synthetically convert the lead candidates to phospholipid prodrugs (Figs 1 and S1). We also hypothesized that these constructs can be self-assembled into well-defined pro-haloacetate nanoparticles. Under this framework, the encapsulation of free drug molecules can be evaded which would allow us to bypass systemic dis-integration and reduce drug-induced off target toxicity. The other potential benefits will also include longer systemic circulation and the ability for enhanced tumor accumulation through passively targeted machinery, resulting in a better efficacy.

### Molecular modelling studies

**Figure 2:**
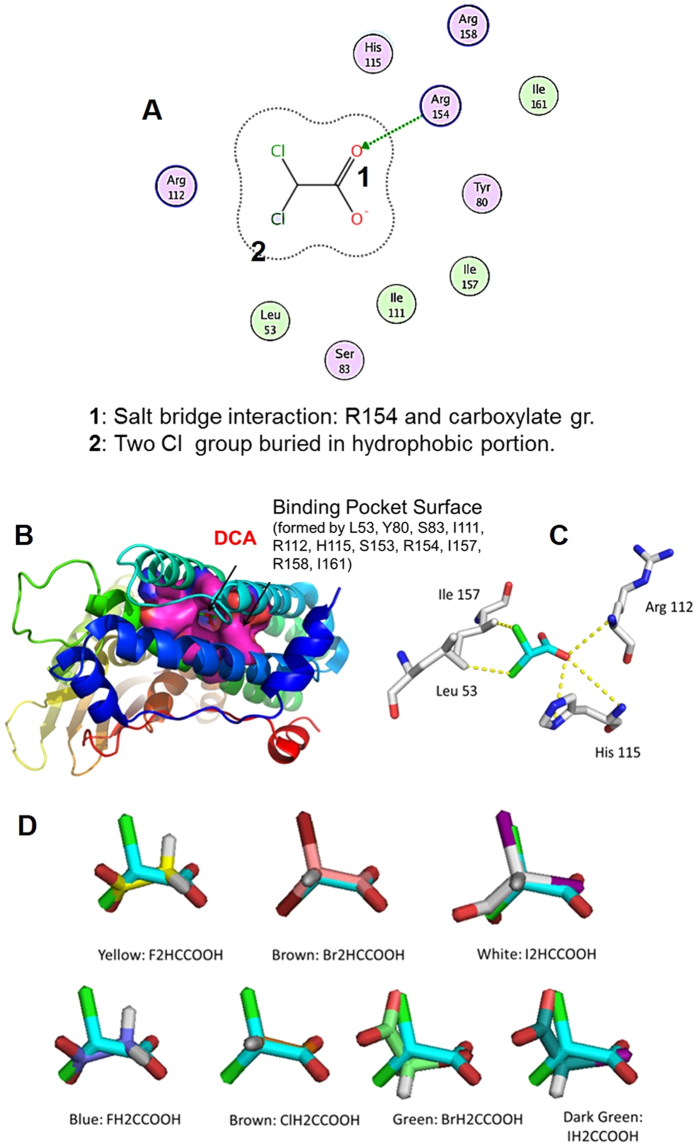
*In-silico* studies to identify lead structure and key interactions. (**A**) Key identified interactions of DCA to the residues of the target DCA binding pocket of 2BU8; (**B**) docking pose of dichloroacetate (Cyan) to binding pocket of 2BU8; (**C**) key interaction of docking pose pose of DCA with the target; (**D**) superimposition of docking pose of various haloacetates, with dichloroacetate (Cyan).

We have studied the molecular recognition of DCA and other structurally related mono- and di- halo (fluoro-, chloro-, bromo- and iodo-) acetic acid-based potential metabolic-modulating agents. The rationale was that the haloacetic acids with varying polarity profiles would lead to selective recognition of PDK isoforms allowing us to identify better haloacetates drug candidates. There are four PDK isoforms expressed in most of the tissues with the maximum sensitivity to DCA being toward PDK2 (PDB code: 2BU8). The DCA binding pocket (Fig. S2) is formed by residues Leu53, Tyr80, Ser83, Ile111, Arg112, His115, Ser153, Arg154, Ile157, Arg158, and Ile161 (Fig. 2A). Most of these residues are conserved among PDHK isoforms from different species. In the docking pose of DCA to the binding pocket of PDK2 target, one oxygen in the carboxylate group of DCA forms a salt-bridge with Arg112, a hydrogen bond interaction with His115, and another oxygen forms a hydrogen bond with Arg158, while the two chlorine substituents are deeply buried in the hydrophobic portion of the pocket to form interaction with Leu53 and Ile157 (Fig. 2B,C).

DCA is sandwiched between His115 and Ile157. In the structure of PDHK3, where a phenylalanine (Phe153) is interchanged in place of Ile157, the DCA binding affinity is greatly diminished due to the Phe side chain occupancy of the pocket. This suggests the existence of the DCA binding cavity in this location of the PDHK1/PDHK2 structure and the importance of these interactions between DCA and key residues. To explore the substitution of other halogen atoms to the chlorine atom, we performed the computational docking with difluoro-, monofluoro-, monochloro-, dibromo-, monobromo-, diiodo- and monoiodoacetates, respectively (Fig. 2D). From Fig. 2D, it could be concluded that docking poses of di-bromoacetate (DBA) and monochloroacetate (MCA) are superimposed with DCA quite well, whereas other molecules show dissimilar docking poses. The quantitative docking energy data from the molecular recognition studies suggested that MCA was a comparable PDK binder while DBA showed a slightly weaker binding than the DCA (Table 1). Based on this observation, DCA, DBA and MCA were selected for further synthetic and biological studies.

### Synthesis of Pro-haloacetates and triggered cleavability

In order to synthetically convert the lead candidates (DCA, MCA and DBA) to phospholipid prodrugs, the haloacetates were subjected to a carbodiimide-mediated esterification modification in presence of 1-palmitoyl-2-hydroxy-sn-glycero-3-phosphocholine (16:0 Lyso PC lipid), EDC and DMAP (67–82% yield) (Fig. S1)^16^. ^1^H-NMR revealed the characteristic proton peaks at δ 5.95, 4.08 and 5.89, respectively for Pro-DCA, Pro-MCA and Pro-DBA. The feasibility of these compounds to undergo enzymatic degradation was studied as prodrugs were allowed to stir for 10 min in pH 7.4 buffer (sodium phosphate buffered saline) in presence of catalytic amount of porcine liver esterase at ambient condition. The enzymatic release of DCA, MCA and DBA were confirmed by HRMS (Supporting information).

### Preparation of pro-haloacetate-NP

**Figure 3:**
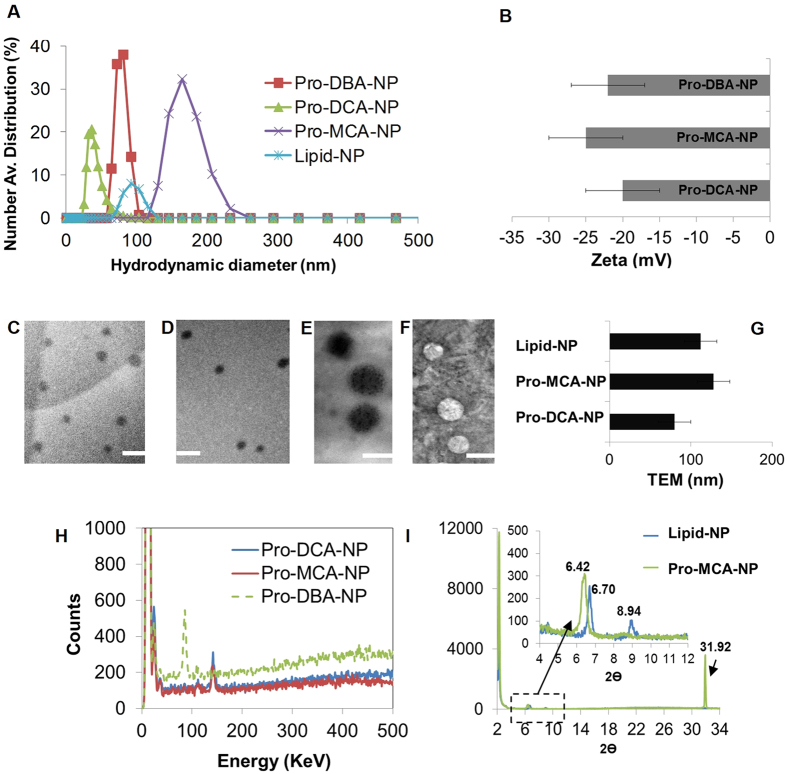
Physico-chemical properties of pro-haloacetate nanoparticles. (**A**) Number avg. hydrodynamic diameter distribution; (**B**) Surface zeta potential distribution; Representative TEM images of (**C**) Pro-DCA-NP; (**D**,**F**) Pro-MCA-NP and (**F**) lipid-NPs (4% uranyl acetate) with comparative anhydrous diameter (**G**) and Representative EDX elemental distribution histograms from (**H**) pro-haloacetate nanoparticles (**I**) representative XRD pattern from lipid-NP and Pro-MCA-NP.

Pro-haloacetate nanoparticles were co-self-assembled *via* solvent evaporation method (H_2_O: THF = 4:1) in presence of polyethyleneglycol cetyl ether (PEGCE). Hydrodynamic diameter of pro-haloacetate nanoparticles was determined by dynamic light scattering (DLS) experiment^17^. The resultant number averaged hydrodynamic diameter distribution of Pro-DCA-NP, Pro-DBA-NP and Pro-MCA-NPs were 38 ± 2 nm, 96 ± 16 nm and 202 ± 33 nm, respectively, while in case of lipid-NPs, it was found as 89 ± 6 nm (Fig. 3A, Table S1). The electrophoretic potential of pro-haloacetate nanoparticles were found to be −20 ± 5, −25 ± 5, and −22 ± 5 mV for Pro-DCA-NP, Pro-MCA-NP and Pro-DBA-NP, while lipid-NP was −35 ± 5 mV (Fig. 3B).

### Physico-chemical characterizations

Transmission electron microscope (TEM) measurements revealed spheroidal nano-particles with characteristic high contrast presumably due to the dense abundance of halogen atoms (Cl and Br) (Fig. 3C–E). The spheroidal particles from Pro-DCA-NP (Fig. 3C) were found to be 80 ± 20 nm, while Pro-MCA-NP (Fig. 3D,E) was a little bigger to produce 128 ± 20 nm particles (Fig. 3G). Evaluation of elemental compositions were acquired by EDX studies while surface topographies were imaged under SEM. Representative EDX spectra for Pro-DCA-NP (Fig. 3H) showed the presence of Cl similar to Pro-MCA-NP (Fig. 3H), while the presence of Br was confirmed in case of Pro-DBA-NP (Fig. 3H). Spheroidal distributions were found in all the cases under SEM too. Interestingly, pro-haloacetate nanoparticles were found to have different d spacing in outer molecular layers in comparison to the lipid-NPs revealing the presence of haloacetic acid prodrug within the lipidic monolayer. Prominent XRD peaks were noticed at 2θ values of 2.24 and 2.26 associated with d spacing of 39.53 and 39.13 Å representing lipid-nanoparticles and Pro-MCA-NP (Fig. 3I, Table 2). When correlated with energy minimized structures of Pro-haloacetates, mono-molecular assemblies were perceived around ‘rigid’ core comprising PEGCE. For this minimization study, the structures of prodrugs and lipids were subjected to all atoms minimization with MOE: 2013. Energy minimization was performed with MMFF94x force field with 0.1 RMS kcal/mol gradients. Energy minimized structures were generally in agreement with the experimental d spacing. The d spacing values were found to be a little larger in case of lipid-NP (39.5) compared to Pro-MCA-NP (39.1), which signified the introduction of large curvature in molecular axis due to pro-drug insertion (Table S2).

### *In-vitro* growth regression studies

**Figure 4:**
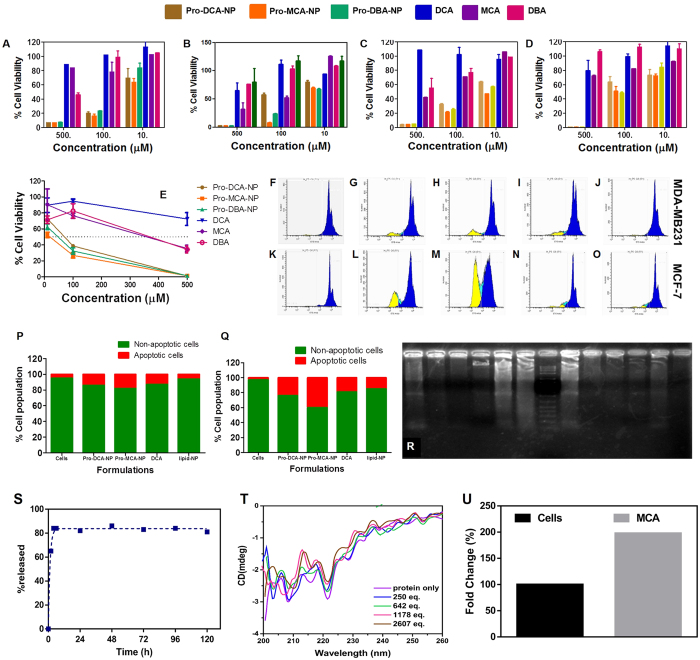
Cell growth inhibition studied by MTT assay. Assay performed in (**A**) Panc1; (**B**) MCF-7; (**C**) MDA-MB231 and (**D**) BT549 cells treated with formulations at concentration ranging from 10–500 μM for 48 h and (**E**) BT549 treated for 72 h. Representative histograms from propidium iodide stained MDA-MB231 and MCF-7 (F-O) for quantifying % apoptotic population (yellow) after treatment with (**G**,**L**) Pro-DCA-NP; (**H,M**) Pro-MCA-NP; (**I**,**N**) DCA and (**J**,**O**) Lipid-NP formulations at 50 μM for 48 h while (**F**,**K**) represent non treated cells. The %apoptotic population compared in (**P**) MDA-MB231 and (**Q**) MCF-7 cells. (**R**) Gel electrophoresis performed on fragmented genomic DNA extracted from MCF-7 (Lane 1–6) and MDA-MB231 (Lane 8–13) cells while lane 7 represent 1 kb ladder. Genomic DNA was extracted from cells treated with (Lane 1, 8) Pro-MCA-NP; (Lane 2, 9) DCA; (Lane 3, 10) untreated cells; (Lane 4, 11) DBA; (Lane 5, 12) MCA and (Lane 6, 13) Pro-DCA-NP formulations at 50 μM in MDA-MB231 and 200 μM in MCF-7 due to different IC50 levels in both the cells for 72 h. (**S**) Drug release study performed on Pro-MCA-NPs against acetate buffer (pH 4.6); (**T**) circular dichroism performed for PDK2 protein against added MCA and (**U**) mitochondrial enrichment of MCA calculated by estimating chloride provide significant input on role of MCA after being released from Pro-MCA-NP.

*In vitro* growth regression was evaluated in Panc1 (pancreatic carcinoma, epithelial-like cell line), BT549 (Estrogen receptor (−) breast cancer invasive ductal carcinoma), MDA-MB231 (ER (−) breast cancer invasive ductal carcinoma) and MCF-7 (ER (+) breast cancer adenocarcinoma) human cells using 3-(4,5-dimethylthiazole-2-yl)-2,5-diphenyltetrazolium bromide (MTT) reduction assay. Interestingly, across all the used cell lines, Pro-MCA-NP was found to be a better cancer cell growth inhibitor compared to Pro-DCA-NP and Pro-DBA-NP. The lipid-NPs were found to have no significant cell growth inhibition capabilities in Panc1 (Fig. 4A), MCF-7 (Fig. 4B), MBA-MB231 (Fig. 4C) and BT549 cells (Fig. 4D) after 48 h incubation and in BT549 cells after 72 h of incubation (Fig. 4E). A more than two order improvement in IC50 was achieved at least in one of the cell lines (i.e. MDA-MB231) specifically in case of Pro-MCA-NP, while the others improved it by 3–10 folds. This clearly underlines the significance of our strategy resulting in an efficacious anti-cancer approach (Table 3). Representative bright field images of BT549 cells show the effect of treatments (48 h, 100 μM) on cell growth density and morphology in monolayer cultures (Fig. S3). Involvement of cellular apoptosis in cell growth inhibition have been followed successfully by determining sub-G1 population *via* propidium iodide (PI) staining assay and DNA laddering assay for fragmented genomic DNA in due course of apoptosis^18^. Cells treated with Pro-MCA-NP were found to be comprising highest % apoptotic cells as 18 ± 2 (Fig. 4H) and 39 ± 4 (Fig. 4M) in MDA-MB231 and MCF-7 cells followed by cells treated with Pro-DCA-NP inducing 14 ± 1 (Fig. 4G) and 24 ± 3% (Fig. 4L) apoptotic cells compared to merely 13 ± 1 (Fig. 4I) and 18 ± 2 (Fig. 4N) in case of small molecule DCA treatment, while no significant effect in Lipid-NP treatment (Fig. 4J,O) compared to untreated cells (Fig. 4F,K), respectively.

### Mechanistic studies and confirmation of halide release, PDK2 interaction and mitochondrial enrichment

In course of apoptotic process, cells treated with pro-haloacetate nanoparticles were found to be most effective in generating DNA ladders^19^ *via* genomic DNA fragmentation (Fig. 4h, Lane 1, 8, 6 and 3) compared to only parent molecule DCA (Lane (3, 10), irrespective of the cell line MCF-7 (Lane 1–6) and MDA-MB231 (Lane 8–13), while Pro-MCA-NP was found as the most effective formulation in generating fragmented genomic DNA *via* cellular apoptotic induction. Thus, apoptotic analyses revealed the better efficiency of MCA compared to DCA and DBA while Pro-MCA-NP was found to be the best among all the used formulations. Pyruvate dehydrogenase (PDH) is responsible for the rate-limiting conversion of pyruvate to acetyl-CoA, while PDK phosphorylates PDH and inhibits its enzymatic activity^20^. DCA inactivates PDK, leading to reactivation of PDH. Thus, enhancement in PDH level inversely correlates with PDK downregulation and in turn tumor regression *via* induction of apoptosis^21^,^22^,^23^.

Release of the haloacetates from pro-haloacetate-NPs was investigated upon exposure to the acidic environment using acetate buffer (pH = 4.6). It is expected that in the acidic environment, the labile ester bond would experience hydrolysis and bond cleavage, resulting in emergence of the free drug. As seen in Fig. 4S, after two hours there is a substantial decrease in the amount of chloride ion indicative of the released drug. Also, it is conceivable that after four hours onwards there was not much significant variation in the release of chloride ion, presumably due to release of the maximum drug within a smaller time period. Approximately 80% of the drug was released in the media after five days.

Interaction of MCA with PDK2 protein was established through *in silico* studies, as well as experimentally with circular dichroism (CD) on PDK2 protein before and after addition of various equivalence of MCA. The alpha helix characteristic region of proteins is usually investigated in the far UV region, hence PDK2-MCA interactions were studied in range of 190 to 250 nm (Fig. 4T). The CD spectra collected from the protein concentration in absence and in presence of MCA showed noticeable change in two representative minima of alpha helix rich protein as dual negative bands at 222 and 208 nm. This shift in the position was essentially indicating the ligand interaction with the protein leading to change in alpha helix of PDK2 protein.

The eventual delivery of haloaceates in cellular mitochondria was required to modulate the activity of PDK2 protein. To confirm the successful delivery of haloacetates in mitochondria, a model study was performed by treating MCF-7 cells with MCA for 4 h before collecting the mitochondrial fraction and quantifying the level of halide (chloride). The level of chloride was compared with the base level abundant in untreated cells and found to be significantly improved (~200%) (Fig. 4U) confirming the additional delivery of chlorides to mitochondria in MCA treated cells.

**Figure 5:**
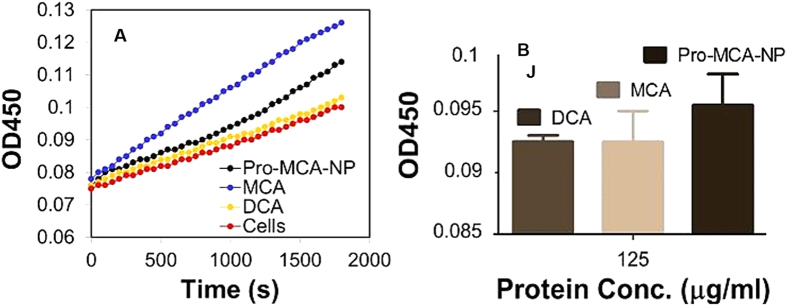
Representative pyruvate dehydrogenase assay on cells. Cells were treated with DCA, MCA and Pro-MCA-NP at 100 μM for 72 h before performing the assay, (**A**) comparative increase in PDH activity of cells correlated with increase in absorbance of reaction mixture at λ 450 nm where “red circle line” represents PDH activity in untreated cells and (**B**) time dependent increase in PDH activity of protein (125 μg/ml).

To verify the PDH modulation efficiencies of newly identified haloacatates and their pro-drug modulated nanoparticles, an assay was performed using manufacturer’s protocol (Abcam; ab109902). Representative PDH enhancement analysis was performed on protein extracted from MCF-7 cells after treatment with MCA and Pro-MCA-NP and compared with DCA treated cells. It was found that Pro-MCA-NP could enhance the PDH level to maximum while small molecule MCA was found better than DCA (Fig. 5A). Time dependent increase in PDH activity of protein was also found to be better for MCA compared to DCA (Fig. 5B). This assay further confirmed that MCA activated PDH to a higher extent compared to well-known PDH activator DCA haloacetate while conversion of haloacetates to pro-drug improved the activity even further.

### Cancer cell selective activity of pro-haloacetate-NPs

**Figure 6:**
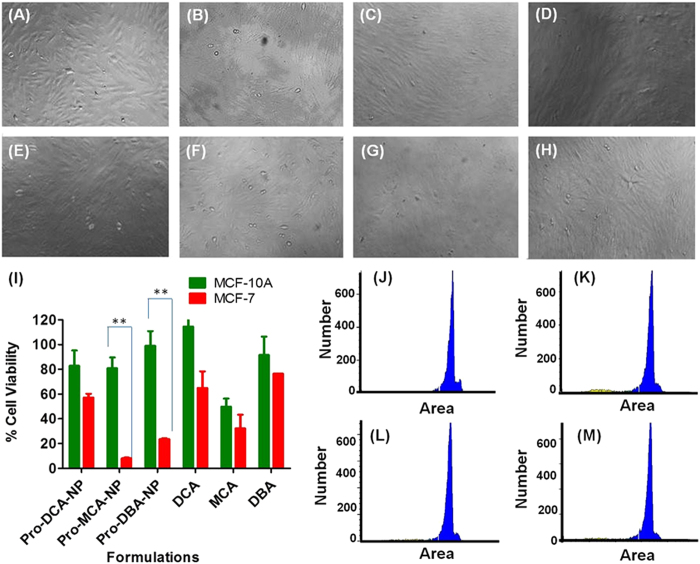
Selective loss of anti-cancer cell growth efficiency of Pro-haloacetate-NPs against non-cancerous cell of breast origin (MCF-10A) compared to cancer cell (MCF-7) of same origin. Bright field imaging performed on (**A**) MCF10A cells post 48 h treatment with (**B**) Lipid-NPs; (**C**) Pro-DCA-NPs; (**D**) Pro-MCA-NPs; (**E**) Pro-DBA-NPs; **(F**) DCA; (**G**) MCA and (**H**) DBA at a concentration of 100 μM revealed no significant loss of cell growth density. (**I**) A comparative cell growth inhibition study in MCF-7 and MCF-10A cells treated with a concentration of 100 μM of DCA, MCA and DBA in free or form of Pro-haloacetate-NPs supporting biostatistically significant low effective toward MCF-10A cells compared to MCF-7 cells by p < 0.01. Propidium iodide incorporation study on (**J)** MCF-10A and treated with (**K**) Pro-DCA-NP; (**L**) Pro-MCA-NP and (**M**) Pro-DBA-NP with a haloacetate concentration of 100 μM for 48 h.

Activity of an anti-cancer formulation improves many folds if it professes the minimal off target toxicity by selective loss in activity against non-cancerous cells. A model cell line of non-cancerous nature and breast origin, MCF-10A was chosen to test the activity of haloacetates and pro-haloacetate-NPs. Bright field imaging was performed on MCF-10A cells after incubation with 100 μM concentration of DCA, MCA and DBA and in nanoparticle form Pro-DCA-NP, Pro-MCA-NP and Pro-DBA-NPs along with untreated or treated with equivalent amount of lipid-NPs. None of the treatments could generate any significant effect on cell morphology and cell growth density revealing selective loss of activity against non-cancerous cells MCF-10A (Fig. 6A–H) compared to cancerous cells MCF-7 (Fig. S3).

MTT assay performed on MCF-10A cells after treatment with 100 μM concentration of MCA and DBA in Pro-MCA-NP and Pro-DBA-NPs exhibited statistically significant difference in cell viability compared to MCF-7 cells. Under the same treatment condition Pro-DCA-NP showed a lower level of selectivity (Fig. 6I). The small molecule haloacetates was found to be selective to a low statistically significant level. To further confirm the ineffectiveness of haloacetates on growth of non-cancerous MCF-10A with no significant upregulation of apoptosis, propidium iodide (PI) incorporation study was performed (Fig. 6J–M). A nominal cell population of less than 5% MCF-10A resulted in an enhanced apoptosis with lower PI staining in fragmented DNA of treated cells by Pro-DCA-NP (Fig. 6K), Pro-MCA-NP (Fig. 6L) and Pro-DBA-NP (Fig. 6M). Thus, all the haloacetate formulations showed selective cancer cell growth inhibition efficiency toward MCF-7 cells compared to MCF-10A with pro-MCA-NPs exhibiting maximum selectivity.

### *In vivo* response of Pro-haloacetate-NPs on cancer regression

**Figure 7:**
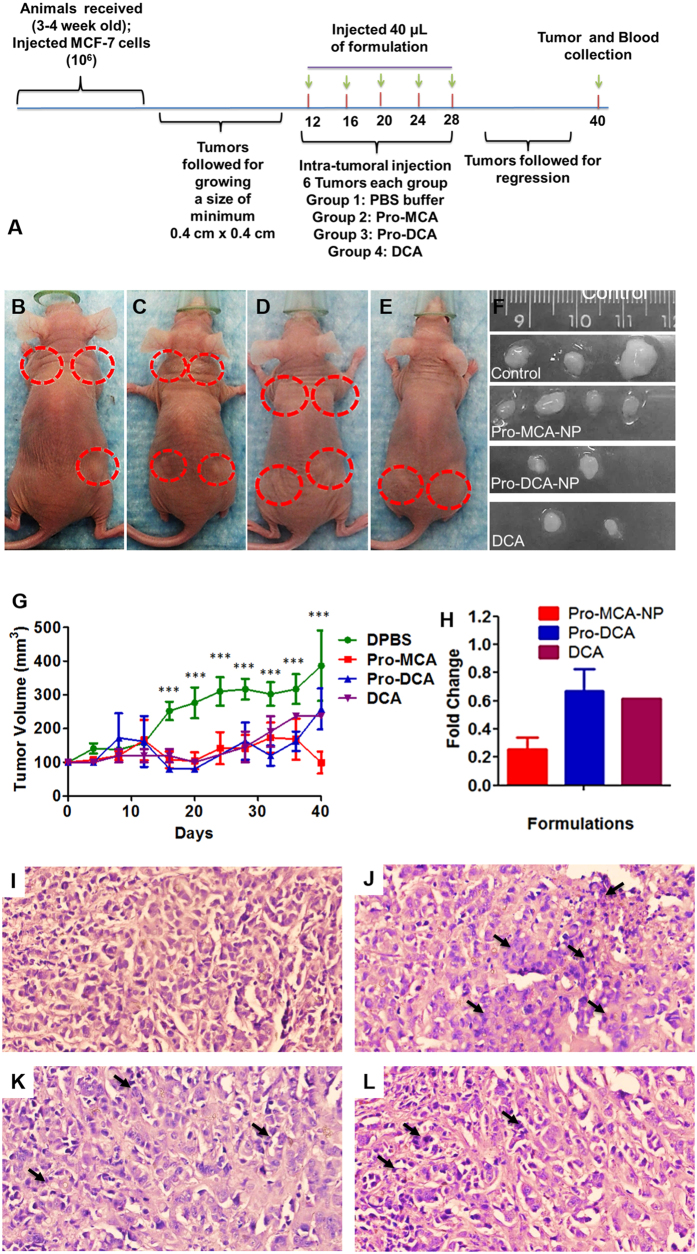
*In vivo* results on xenograft mouse model. (**A**) Timeline of experiment; representative animals with tumors after treatment with (**B**) DPBS; (**C**) Pro-MCA-NP; (**D**) Pro-DCA-NP and (**E**) DCA and **(F**) representative tumors collected after sacrificing the animals. (**G**) Tumour growth curves with time and (**H**) fold change after treatment with Pro-MCA-NP, Pro-DCA-NP and DCA. H&E images of tumour sections for treatments with (**I**) DPBS; (**J**) Pro-MCA-NP; (**K**) Pro-DCA-NP and (**L**) DCA (Black arrows indicate presence of fragmented nuclei in tumor sections).

The tumour regression ability of Pro-MCA-NP, Pro-DCA-NP and DCA was verified *in vivo* in xenograft tumours generated from MCF-7 cells grown in athymic nude mice^24^,^25^. Tumors grown to a minimum size of 0.4 × 0.4 cm^2^ (Fig. 7A) were intra-tumourally injected with phosphate buffer DPBS (Fig. 7B); Pro-MCA-NP (Fig. 7C); Pro-DCA-NP (Fig. 7D) and DCA (Fig. 7E) in 40 μL volume on day 12, 16, 20, 24 and 28 following the first measurement after cell implantation. The growth was followed till 40 days before sacrificing animal and excising the tumour tissue. The regression of tumour growth was found to be biologically significant for Pro-MCA-NP, Pro-DCA and DCA (Fig. 7F) compared to tumours treated with buffer in same order (Fig. 7D). The buffer treated tumours were continued to grow as high as 400% (Fig. 7G) compared to the reduction in size for Pro-MCA-NP treatment to as low as 40%. The tumour regression was found maximum for Pro-MCA-NP with fold change of 0.4 compared to 0.8 for Pro-DCA-NP and DCA treatments (Fig. 7H). Interestingly, DCA and Pro-DCA-NP formulations did not show statistically significant difference in tumor regression at the end of the study. However, a close analysis revealed that Pro-DCA was more effective in regression at earlier time points than DCA. On the contrary, Pro-MCA was found to be more effective throughout the study period confirming MCA being more efficacious in tumor regression in comparison to DCA. We anticipate that DCA mediated treatments required higher amount of doses of treatment to trigger more sustained regression of tumor with no apparent resurrection during the withdrawal period.

*In vivo* results were corroborated with histologic analyses. Hematoxylin/eosin (H&E) stained sections of tumors revealed nuclear fragmentation and retracted cytoplasm indicating occurrence of apoptotic features in tumour sections treated with haloacetate-NPs (Fig. 7J–L).

## Conclusions

In summary, this work proposes a strategy for the first time to deliver potent, PDK-modulating haloacetic acids in pro-drugs encompassed in a clinically translatable nanoplatform. These agents are inexpensive and selectively inhibit PDK for reversing the suppression of mitochondria-dependent apoptosis. Administration of the agent in a ‘protected’ prodrug form, by virtue of requiring a lower administered dose will reduce systemic toxicity while simultaneously enhancing drug uptake. This will permit use of lower drug dosages, reducing overall toxicity while increasing overall efficacy. If successful, this approach could offer new hope for cancer patients with much desired improvements in their treatment regimen with affordable cancer therapeutics.

## Materials and Methods

DCA, MCA, DBA were obtained from Sigma Aldrich, Inc. (St. Louis, MO) while LysoPC was purchased from Avanti Polar Lipids (Birmingham, Al). The hydrodynamic diameter was measured on a Malvern Zetasizer machine equipped with 633 nm laser. Zeta potential measurement was performed on a Malvern Zetasizer instrument, from MRL facility, UIUC. The TEM images were acquired on a JEOL 2100 Cryo TEM machine and imaged by Gatan UltraScan 2k × 2k CCD. The XRD data was collected on instrument Siemens-Bruker D5000 diffractometer and analyzed using software Jade X-ray analysis. MTT reduction assay was ended with performing absorption assay on plate reader (Synergy HT, Bio-Tek). Bright field imaging was performed on microscope DMI3000 B, Leica Microsystems, Buffalo Grove, IL.

### Molecular modeling

DCA, MCA and DBA were built using a SKETCH module in Sybyl-X 2.0. Energy minimization was performed with the Tripos force field until a gradient convergence of 0.05 kcal/mol was achieved. The NB Cutoff was set to 8.00. Distance was set to the dielectric function, and the diaelectric constant was set to 1.00. Small molecules were docked into 2BU8 active site with MOE 2013.08 program. Hydrogens were added to the protein using the ‘Add Hydrogens’ function in MOE. L53, Y80, S83, I111, R112, H115, S153, R154, I157, R158, I161 were chosen as the active site. Induced Fit was utilized as the docking protocol. Triangle Matcher, which generated poses by aligning ligand triplets of atoms on triplets of alpha spheres, was used as the placement method with default settings. London dG was used as the score function. The conformation with the lowest docking energy was chosen as the docking pose for each compound^26^.

### Synthesis of halogen acetate prodrugs

Halogen acetates (4 eq), 1-palmitoyl-2-hydroxy-sn-glycero-3-phosphocholine (16:0 Lyso PC) (1 eq), EDC (2 eq) and DMAP (2 eq) in CHCl_3_ solution was stirred up for 12 h at room temperature. The solution was then concentrated and dried over vacuum to get halogen acetate prodrugs.

#### Dichloroacatate prodrug (Pro-DCA)

^1^H-NMR(CDCl_3_, 500 MHz): δ 8.20 (d, J = 5.0 Hz, 5H), 6.76 (d, J = 5.0 Hz, 4H). 5.96 (s, 2H) 4.39 (m, 2H), 4.11 (m, 1H), 3.92 (d, 4H), 3.43 (m, 8H), 3.32 (m, 9H), 3.24 (s, 15H), 317 (m, 15H), 2.83 (m, 19H), 2.30 (m, 2H), 1.98 (m, 6H), 1.25(m, 34H), 1.10 (m, 9H), 0.86 (m, 12H). HRMS m/z: 606.2724 (MH)^+^, calcd for C_26_H_51_Cl_2_NO_8_P: 606.2729.

#### Monochloroacatate prodrug (Pro-MCA)

^1^H-NMR(CDCl_3_, 500 MHz): δ 8.27 (d, J = 5.0 Hz, 3H), 6.76 (d, J = 5.0 Hz, 5H). 4.38 (m, 3H), 4.22 (m, 2H), 4.09 (m, 3H), 4.02 (m, 1H), 3.95 (m, 2H), 3.41 (m, 9H), 3.31 (m, 7H), 3.25 (s, 10H), 316 (m, 9H), 2.83 (m, 12H), 2.31 (m, 2H), 1.99 (m, 4H), 1.28(m, 24H), 1.09 (m, 7H), 0.87 (m, 4H). HRMS m/z: 572.3107 (MH)^+^, calcd for C_26_H_52_ClNO_8_P: 572.3119.

#### Dibromoacatate prodrug (Pro-DBA)

^1^H-NMR (CDCl_3_, 500 MHz): δ 8.24 (d, J = 5.0 Hz, 4H), 6.76 (d, J = 5.0 Hz, 4H). 5.89 (s, 2H) 4.46 (m, 2H), 4.12 (d, 2H), 4.0 (m, 4H), 3.44 (m, 9H), 3.32 (m, 4H), 3.24 (s, 12H), 318 (m, 8H), 2.84 (m, 12H), 2.78 (s, 1H), 2.31 (m, 2H), 1.99 (m, 4H), 1.58 (m, 2H), 1.24(m, 24H), 1.11 (m, 6H), 0.85 (m, 4H). HRMS m/z: 694.1727 (MH)^+^, calcd for C_26_H_51_Br_2_NO_8_P: 694.1732.

##### Acidic pH cleavage of haloacetate prodrugs

We determined whether the pro-haloacetates can be degraded in the presence of acidic pH. The prodrug (500 μL) was incubated for 2 h with acidic pH (4.1). The samples were continuously agitated on a nutator to prevent settling. The mixture was isolated and analyzed by HRMS to observe signature peaks of cleaved and liberated haloacetates. High-resolution mass spectrometry (HRMS) confirmed the release of haloacetates from the prodrug under acidic pH condition.

Dichloroacetate (DCA): HRMS m/z: 126.9355 (M-H)^+^, calcd for C_2_HCl_2_O_2_: 126.9354; Monochloroacetate (MCA): HRMS m/z: 92.9745 (M-H)^+^, calcd for C_2_H_2_ClO_2_: 92.9743; Dibromoacetate prodrug (DBA): HRMS m/z: 214.8346 (M-H)^+^, calcd for C_2_HBr_2_O_2_: 214.8343.

### Enzyme catalyzed cleavage of haloacetate prodrugs

We also determined whether the pro-haloacetates can be degraded in the presence of esterases from porcine liver. The prodrug (500 μL) was incubated for 30 min with porcine liver esterase (1u). The samples were continuously agitated on a nutator to prevent settling. The mixture was isolated and analyzed by HRMS to observe signature peaks of cleaved and liberated haloacetates. High-resolution mass spectrometry (HRMS) confirmed the release of haloacetates from the prodrug under acidic pH condition.

Dichloroacetate (DCA): HRMS m/z: 126.9353 (M-H)^+^, calcd for C_2_HCl_2_O_2_: 126.9354; Monochloroacetate (MCA): HRMS m/z: 92.9745 (M-H)^+^, calcd for C_2_H_2_ClO_2_: 92.9743; Dibromoacetate (Pro-DBA): HRMS m/z: 214.8344 (M-H)^+^, calcd for C_2_HBr_2_O_2_: 214.8343.

### Preparation of Pro-Drug-NPs

Polyethylene glycol cetyl ether (0.5 mg) was melted at 65 °C for 5 min followed by the dropwise addition of 1 ml of water (approximately 1 drop/sec). The solution was allowed to stir for 20 min at 1150 rpm. A THF solution of Pro-DCA, Pro-MCA and Pro-DBA (250 μL) was added drop-wise (approximately 1 drop/10 sec) to the aqueous suspension of Polyethylene glycol cetyl ether. Preparations were also performed using polyethylene glycol cetyl ether (1 mg) and THF solution of Pro-DCA, Pro-MCA and Pro-DBA (varying to 500 and 125 μL). The solution was left for stirring overnight to allow THF evaporation. At the end of the procedure, volume was made up to 1 mL with autoclaved nanopure water (0.2 μM). The suspension was further allowed to stir for 10 min at room temperature. Finally, the suspension was stored at 4 °C overnight for curing the core of the particle and size measurement was performed. The nano particles were purified by dialysis against nanopure (0.2 μm) water using a 10,000 Da MWCO cellulose membrane for prolonged period of time. The nanoparticles were stored under argon atmosphere typically at 4 °C in order to prevent any bacterial growth. The shelf-life stability of the prepared particles were found to be remarkably high for the combination of polyethylene glycol cetyl ether (0.5 mg) and THF solution of Pro-DCA, Pro-MCA and Pro-DBA (250 μL). Particles prepared from other combinations were found highly unstable and found to aggregate within 24 h of storage at 4 °C.

### Dynamic light scattering measurements

Hydrodynamic diameter distribution and distribution averages for the Pro-Drug-NPs of Pro-DCA-NP, Pro-MCA-NP and Pro-DBA-NP were determined by dynamic light scattering. Hydrodynamic diameters were determined using a Malvern Zeta Sizer ZS90 particle size analyzer. Measurements were made following dialysis (MWCO 10 kDa dialysis cassette, Thermo Scientific) of nanoparticle suspensions into deionized water (0.2 μM). Scattered light was collected at a fixed angle of 90°. A photomultiplier aperture of 400 mm was used, and the incident laser power was adjusted to obtain a photon counting rate between 200 and 300 kcps. Only measurements for which the measured and calculated baselines of the intensity autocorrelation function agreed to within +0.1% were used to calculate nanoparticle hydrodynamic diameter values. All determinations were made in multiples of five consecutive measurements.

### Electrophoretic Potential Measurements (Zeta Potential)

A nano series Malvern Zetasizer zeta potential analyzer was used to collect zeta potential (ζ) values of the Pro-Drug-NPs. Data was collected using the in the phase analysis light scattering (PALS) mode at 25 °C. The ζ value was derived from calculation of the electrophoretic mobility (μ) using the Smoluchowski equation: μ = ε/η, where ε is the dielectric constant and η is the absolute viscosity of the medium. Five determinations of ten (10) data accumulations were reproducible within ±5.0 of the mean value^27^.

### Cast-film X-Ray Diffraction Measurement

The layered arrangements and variations in inter-layer spacing of outer shells of Pro-Drug-NPs and Lipid-NPs were determined by X-ray diffraction (XRD) measurement. The mixed micelle of each formulation was placed on a pre-cleaned glass plate which, upon air drying, afforded a thin film of the formulation. XRD of an individual cast film was performed using the reflection method with a Siemens-Bruker D5000 diffractometer. The X-ray beam was generated with a Cu anode and the Cu-Kα beam of wavelength 1.5418 Å was used for the experiments. Scans were performed for 2θ range of 2 to 35^28^.

### Transmission Electron Microscopy

An aliquot of Pro-drug-NP and Lipid-NP was drop-casted onto a carbon coated copper grid (200 mesh size). Lipid-NP samples were stained with 10 μl of uranyl acetate (1%). TEM images were taken on JEOL 2100 Cryo TEM machine and imaged by Gatan UltraScan 2k × 2k CCD. The concentrations were used as Pro-drug-NP containing 400 μM of Pro-Drug molecules and equivalents of LysoPC^29^.

### Scanning Electron Microscopy and EDX for elemental studies

Scanning electron microscopic studies were performed on Pro-drug-NP to evaluate surface properties of their cluster. Additional Energy dispersive X-ray (EDX) spectrum analysis was also performed to demonstrate the presence of halogen, N and P elements in addition to C and O in Pro-drug-NP preparations. Samples were prepared on metal stubs covered with carbon tape. Suspension of Pro-drug-NP containing 1 mM of prodrug molecules were used to drop cast on carbon tape and allowed to air-dry before putting in vacuum for 5 h^30^.

SEM images were taken on Hitachi S-4700 High Resolution SEM machine with capabilities of high resolution low voltage imaging and connected to cold field emission gun (2.5 nm resolution at 1 kV, 1.5 nm resolution at 15 kV) while EDX was performed on ISIS EDS X-ray Microanalysis System (Oxford Instruments) using in build software.

### MTT Assay and cell morphological investigations

Cancer cell growth regression was evaluated in Panc1 (Human pancreatic carcinoma, epithelial-like cell line); BT549 (ER (−) Human breast cancer invasive ductal carcinoma); MDA-MB231 (ER (−) Human breast cancer invasive ductal carcinoma) and MCF-7 (ER (+) Human breast cancer adenocarcinoma) cells. The cell viability efficiency of Pro-drug-NPs along with parent small molecules DCA, MCA and DBA formulations in cell lines of different origin were performed using 3-(4,5-dimethylthiazole-2-yl)-2,5-diphenyltetrazolium bromide (MTT) reduction assay^31^,^32^. Assay was performed in presence of 10% FBS in antibiotic free media. Experiment was performed in 96 well plates (CELLSTAR; Germany) growing 10,000 cells per well for 24 h before treatments. Experiments were performed for various concentrations of DCA, MCA and DBA in parent or Pro-drug-NP form varying from 10 and 500 μM. Cells were incubated for 48 h and 72 h before performing the MTT assay. Cell morphology was monitored by bright field imaging. After incubation period, cells were treated with MTT as 20 μL (5 mg/mL) per well and further incubated for 4 h. At the end of the incubation entire medium was removed from wells and 200 μL DMSO was added to dissolve blue colored formazan crystals. The percentage cell viability was obtained from plate reader and was calculated using the formula % cell Viability = {[A592(treated cells) − (background)]/[A592(untreated cells) − background]} × 100.

### Propidium iodide staining assay for cellular apoptosis

Cells were treated with Pro-drug-NP and haloacetates followed by flow assisted cell sorting (FACS) analysis on propidium iodide (PI) stained treated and untreated cells. Results were analysed to calculate the apoptotic cell population^33^,^34^. Cells (0.3 × 10^6^ per well) were plated in 6 well plates and grown till it achieved ~80% confluence. After ~24 h of incubation, cells were treated with 50 μM of haloacetates in free or form of Pro-drug-NP. At the end of 72 h time point, cells were imaged under bright field microscope for cell growth density and morphological determination. Cells were trypsinized and collected in 100 μl of reconstituted medium (DMEM containing 10% FBS). Cell pellets were fixed with chilled ethanol while vortexing to homogenize the samples. Fixed cells were stored at −20 °C for >12 h. At the end of the incubation, cells were washed with DPBS at least two times and incubated with RNase A (1 μg/mL) at 37 °C for >12 h. Cells were incubated with PI (2 μg/mL) for 30 min before scanning on FACS machine. Cells were treated in triplicated and pooled down before acquiring the FACS data.

### Laddering assay for fragmented genomic DNA post apoptosis

DNA fragmentation assay^35^ was performed to further probe cell apoptosis as described earlier. Briefly, 24 h grown cells were treated with Pro-drug-NP and haloacetates at 50 μM in MDA-MB231 and 200 μM in MCF-7 due to different IC50 levels in both the cells for 72 h before harvesting in 1 mL of 10% FBS containing culture medium at the end of the incubation period and washed with 1 mL DPBS two times. Harvested cells were spun at 1000 rpm for 2 min, to obtain cell pellets. Cells pellet were trypsinized and collected in 400 μL of lysis buffer. DNA extraction was performed using manufacturer’s protocol using Thermo Scientific DNA extraction kit. Extracted genomic DNA were washed with 70% EtOH and dissolved in water after air drying. Collected genomic DNA was used to load in 2% agarose gel and ran at 100 mV for 40 min before imaging on Gel doc.

### Drug release studies

The drug release profile was monitored at pH 4.6 using acetate buffer (CAS No. 126-96-5, Sigma, St Louis, MO) for 120 hours. An aliquot of 100 μL from 5 mM DCA sample was pipetted into Slide-A-Lyzer^TM^ mini dialysis device (10 K MWCO, 0.1 ml, Thermo Scientific, IL, USA) and dipped into 1 L of the buffer media to simulate sink condition. The buffer media was magnetically stirred (120 rpm) to keep the conditions homogenous across the solution while keeping the temperature constant at 37 °C. At the indicated time points, 45 μL of sample aliquot was recovered and was diluted 10× for further quantitative analysis via Ion Selective Electrode method. Control for the drug release study was a prodrug nanoparticle at time t = 0 h and its concentration was considered as 100% compared to released drug concentration after various time points of drug release.

### Circular Dichroism (CD) studies

CD spectroscopy is generally used to study chirality of molecules, especially, protein folding and interaction in aqueous media due to the facile manipulation of the experimental conditions and its high sensitivity towards minute variations in temperature, pH and amphiphiles. The protein’s native CD spectra and the changes upon drug titration were obtained using a Jasco 715 spectro-polarimeter (Mary’s Court Easton, MD, USA) and using a quartz cuvette (Starna Cells, Atascadero, CA) with 2 mm path length. All spectra were acquired from 200 to 260 nm and are indicate as the average of three accumulations with the scanning resolution of 0.5 nm. A 50 μL of PDK2 protein (50 μg at 1.5 mg/mL) was transferred to the cuvette with 500 μL of PBS (1×) and 1 μL of monochloroacetic acid (25 mM in DI water) was titrated to the cuvette at each addition. The drug was mixed properly and was incubated three minutes before data acquisition. The titration was terminated when no noticeable change was observed in the spectra pattern. The measured curves were smoothed using Savitzky- Golay algorithm by taking 10 points fitting a second order polynomial curve.

### Determination of delivered drug in mitochondrial fraction

Haloacetates (DCA, MCA and DBA) were established to interact with mitochondrial protein PDK2 *in silico*, while interactions in intracellular space was warranted to support the claim. Strategy was employed to check the enrichment of haloacetate in mitochondrial fraction of haloacetate treated cells to establish the fact that eventually haloacetate being delivered to cellular mitochondria. The cell fractionation was performed on MCF-7 cells untreated or treated with 100 μM of MCA for 4 h before collecting the mitochondrial fraction using literature protocol. Briefly, Cells were trypsinized and pelleted down in 1 ml of DPBS buffer. Cells were homogenized under probe sonicator at the setting of Amp 1 for 10 sec at RT. Cellular homogenate was filtered through cellulose filter to remove unbroken cells. Then homogenate was centrifuged at a centrifugal force of 600 g (600 times the force of gravity) to sediment nuclei; followed by centrifuging the supernatant at a higher speed of 15,000 g for 5 min, sedimenting mitochondria, chloroplasts, lysosomes,and peroxisomes. Mitrochondrial fractions were collected and used for determining haloacetate meditaed enhancement in halide level, a directly proportional trait to delivery of MCA in mitochondria of treated cells.

### Determination of the halide concentration

The concentration of halide i.e. Cl^−^ was investigated using Thermo Scientific Chloride Ion Selective Electrode (ISE) by the calibration curve method (13-641-877 Thermo Scientific Chloride standard). Five points calibration solution were prepared in order to construct the corresponding calibration curves. The method relies on the direct potentiometry for the determination of analyte in the system. Careful attention was paid to the material, temperature and stir speed and enough time was allowed for the reaching equilibration prior to measurement. For the samples collected from the cell fractions, the combustion method was applied where the halide gas was absorbed into a liquid, which is then used for the ISE analysis. The samples obtained from the kinetics of release studies were directly pipetted into a volumetric flask and then diluted with deionized water to create the solutions to be analyzed.

### Cancer cell selective activity of Pro-haloacetate-NPs

To establish the selective activity of prepared Pro-haloacetate-NPs, bright field imaging, cell viability assay and propidium iodide incorporation assays were performed on non-cancerous cells of breast cancer origin, MCF-10A and compared it with cancerous cells MCF-7 of same origin. MCF-10A cells (10 × 10^3^) were grown in 96 well plate for 24 h before treating with 100 μM concentration of DCA, MCA and DBA in free or form of pro-haloacetate-NPs. At the end of the post incubation of 48 h, cells were imaged under bright field and used for MTT assay as discussed earlier. For propidium iodide incorporation asaay, MCF-10A cells (4 × 10^5^) grown in 6 well plate for 24 h before treating with 100 μM concentration of Pro-DCA-NP, Pro-MCA-NP and Pro-DBA-NP. After 48 h of incubation cells were fixed with 75% ethanol and stained with propidium iodide (10 μg/mL) for 30 min before performing the FACS analysis.

### Pyruvate dehydrogenase activity assay and down regulation of PDKs

A pyruvate dehydrogenase (PDH) enzyme activity microplate assay kit (Abcam; ab109902) was used to evaluate PDH activity of haloacetate^36^ and Pro-haloacetate-NPs. Cells (10 × 10^6^) were grown to 80% confluency in 60-mm dish for 24 h before treating with 50 μM concentration of haloacetate in free or form of Pro-drug-NP. After 72 h of treatments, cells were placed into ice and washed with ice-cold PBS. Protocol for protein extraction was used as supplied by Minute^TM^ Total Protein Extraction Kit for Animal Cultured Cells and Tissues (Invent Biotechnologies, Inc.). Protein quantification was achieved by using manufacturers’ protocol supplied by Protein Quantification Kit-Rapid (Sigma-Aldrich Inc.). Quantified protein was used for PDH assay using manufacturer’s protocol (Abcam; ab 10992).

### Animal studies

While advanced 3D cell culture techniques can mimic some of the aspects of the *in vivo* tumor environment, these techniques are still lacking in the complexity found *in vivo*. In addition to tumor architecture, the global setting of the tumor in the animal cannot yet be replicated with cell culture techniques. Thus, after optimizing treatment strategies *in vitro*, treatments have to be established in animal models.

The experimental protocol was approved by the Institutional Animal Care and Use Committee (IACUC), University of Illinois, Urbana–Champaign, and satisfied all University and National Institutes of Health (NIH) rules for the humane use of laboratory animals. All the animal experiments were carried out in accordance with the approved guidelines.

To evaluate the efficacy of adopted strategy using lipid conjugated haloacetates, animal experiments were performed. All experiments were designed to minimize the use of animals. In order to detect a least of 20% difference in tumor size, we decided to generate 4 tumors per animal as 3 mice per group. Athymic mice were bought from Charles River Laboratories International, Inc. USA. Upon arrival, athymic mice were allowed 1 week for acclimation. Animals were single-cage housed and had free access to food and water. Animals were housed in Carl R. Woese Institute for Genomic Biology, university of Illinois at Urbana-Champaign.

### Injection of MCF-7 (ER(+) human breast cancer cells) in flanks of anthymic mice

Animals were anesthetized with isoflurane before injecting the MCF-7 cells suspended in Matrigel (50%, v/v). Using a Hamilton auto-injector syringe tipped with a 26 gauge 1/2′′ long needle, we subcutaneously injected approximately 5 × 10^6^ MCF-7 human breast cancer cells^37^ suspended in 40 μL of Matrigel into four sites in the flank of each mouse. Mice were monitored during recovery from the anesthesia in a clean cage. MCF-7 tumors were grown on the back of mice after cell injection. We did not find that in the time frame of completing the experiment grown tumors caused any significant discomfort to the mice. We monitored the mice daily for signs of discomfort and behavior change. Mice body weight was measured every week. The change in physiological function or abnormal behavior including shortness of breath, unsteady gait, abnormal eating behavior, physical abnormalities, rough hair coat due to lack of grooming, or lethargy were reported to division of animal research. Criteria for interventions were set up as animal body weight drops by 20% or tumor increase to 17 mm × 17 mm. Tumor size was determined by measuring the length and width of the tumor and then calculating the tumor volume *via* formulae^38^.


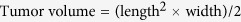


### Intra-tumoral injections

Animals were followed till tumors grew to a minimum of 4 mm × 4 mm before starting the treatment protocol. Pro-MCA-NP and Pro-DCA-NP were prepared at 5 mM concentration of MCA and DCA and injected to tumors grown to at least 4 mm × 4 mm dimensions. Isoflurane-Oxygen mixture was used to anesthetize the animals with 3–4% isoflurane gas from a vaporizer and constant anesthesia maintained with 1–2% isoflurane via an inlet tube. A second tube was used to remove carbon dioxide and excess anesthetic. All the personnel involved wore protective lab coats, face masks, sterile gloves during experimental procedures. A total of 40 μL formulation were injected to each tumor on every 4 th day including 12, 16, 20, 24 and 28 th day and followed till 40th day for tumor growth and regression^39^.

### Animal dissection, tumor collection, processing, embedding and sectioning

At the end of the experiment, animals were euthanized with CO_2_ influx. Animals were dissected to collect tumors and stored in tissue cassettes dipped in 10% formalin before performing the tissue fixation protocol in Leica ASP300 tissue processor. The processing protocol was used including steps of tissue incubation in neutral buffered saline for 45 min, twice with ethanol (70%) for 45 min, ethanol (80%) for 45 min, twice with ethanol (95%) for 45 min, twice with absolute ethanol for 45 min, twice with xylene for 45 min and finally thrice with paraffin wax for 45 min. Processed tumors were embedded in paraffin wax melted at 65 °C using metal cast. Embedded tumor blocks were clamped in microtome (Leica) and sectioned at 6 μm thickness.

### H&E staining

Paraffin-embedded sections (7 μm) were subjected to hematoxylin and eosin (H&E) staining^40^. H&E staining was performed by following standard protocol supplied at core facility IGB. In brief, sections were processed by deparaffinize sections with 3 changes of xylene for 5 minutes each; re-hydrate in 3 changes of absolute alcohol for 5 minutes each; 95% alcohol for 3 minutes and 70% alcohol for 3 minutes each; wash briefly in distilled water; stain in Mayer hematoxylin solution for 3 minutes; wash in warm running tap water for 5 minutes; rinse in distilled water; rinse in 95% alcohol, 10 dips; counterstain in eosin-phloxine B solution (or eosin Y solution) for 3 minute; dehydrate through 95% alcohol, 2 changes of absolute alcohol, 5 minutes each; clear in 2 changes of xylene, 5 minutes each; mount with xylene based mounting medium. The morphological changes of H&E-stained tissue with each fixation were analyzed at magnification x40.

## Additional Information

**How to cite this article**: Misra, S. K. *et al*. Pro-haloacetate Nanoparticles for Efficient Cancer Therapy *via* Pyruvate Dehydrogenase Kinase Modulation. *Sci. Rep.* **6**, 28196; doi: 10.1038/srep28196 (2016).

## Supplementary Material

Supplementary Information

## Figures and Tables

**Table 1:** The quantitative docking energy data from the molecular recognition studies of haloacetates.

DBA	DCA	MCA
−3.463 (KJ Mol^−1^)	−4.104 (KJ Mol^−1^)	−4.121 (KJ Mol^−1^)

**Table 2:** Representative 2θ values and d spacing in lipid-NP and Pro-MCA-NP.

Lipid-NP		Pro-MCA-NP	
2θ	d (nm)	2θ	d (nm)
2.24	39.53333	2.26	39.13198
6.70	13.20034	6.42	13.7907
8.94	9.896021	31.92	2.804292

**Table 3:** IC50 values for different formulations across different cell lines.

Cancer Cells	IC50 (μM)					
	Pro-DCA-NP	Pro-MCA-NP	Pro-DBA-NP	DCA	MCA	DBA
Panc-1	45 ± 05	35 ± 05	60 ± 06	≫500	>500	450 ± 45
MCF-7	150 ± 15	40 ± 05	45 ± 05	≫500	140 ± 15	>500
MDA-MB231	50 ± 05	10 ± 05	30 ± 05	≫500	400 ± 50	>500
BT549	180 ± 20	100 ± 10	100 ± 10	≫500	>500	>500

## References

[b1] Bonnet S. . A mitochondria-K+ channel axis is suppressed in cancer and its normalization promotes apoptosis and inhibits cancer growth. Cancer Cell 11, 37–51 (2007). 17222789 10.1016/j.ccr.2006.10.020

[b2] Welsh S., Williams R., Kirkpatrick L. & Powis G. Antitumor activity and pharmacodynamic properties of PX-478, an inhibitor of hypoxia-inducible factor-1alpha. Mol. Cancer Ther. 3, 233–244 (2004). 15026543

[b3] Deus C. M., Coelho A. R., Serafim T. L. & Oliveira P. J. Targeting mitochondrial function for the treatment of breast cancer. Fut. Med. Chem. 6, 1499–1513 (2014). 10.4155/fmc.14.100 25365234

[b4] Gopinath S. & Evangelos M. D. Pyruvate dehydrogenase kinase as a novel therapeutic target in oncology. Front. Oncol. 3, 38 (2013). 23471124 10.3389/fonc.2013.00038 PMC3590642

[b5] Ruggieri V. . Dichloroacetate, a selective mitochondria-targeting drug for oral squamous cell carcinoma: a metabolic perspective of treatment. Oncotarget. 6, 1217–1230 (2015). 25544754 10.18632/oncotarget.2721 PMC4359228

[b6] Srinivasan S., Guha M. & Avadhani N. G. Dysfunctional mitochondria: therapeutic targets in ischemia reperfusion injury and cancer. Mitochond. Dysfun. 1, 1–37 (2014).

[b7] Gong F. . Dichloroacetate induces protective autophagy in LoVo cells: involvement of cathepsin D/thioredoxin-like protein 1 and Akt-mTOR-mediated signaling. Cell Death Dis. 4, e913 (2013). 24201812 10.1038/cddis.2013.438 PMC3847316

[b8] Shen Y.-C. . Activating oxidative phosphorylation by a pyruvate dehydrogenase kinase inhibitor overcomes sorafenib resistance of hepatocellular carcinoma. British J. Can. 108, 72–81 (2013). 10.1038/bjc.2012.559 PMC3553537 23257894

[b9] Kato M., Li J., Chuang J. L. & Chuang D. T. Distinct structural mechanisms for inhibition of pyruvate dehydrogenase kinase isoforms by AZD7545, dichloroacetate, and radicicol. Structure 15, 992–1004 (2007). 17683942 10.1016/j.str.2007.07.001 PMC2871385

[b10] Michelakis E. D. . Metabolic modulation of glioblastoma with dichloroacetate. Sci. Trans. Med. 2, 31–34 (2010). 10.1126/scitranslmed.3000677 20463368

[b11] Wong J. Y. Y., Huggins G. S., Debidda M. & Munshi N. C. *De-vivo*, I. Dichloroacetate induces apoptosis in endometrial cancer cells. Gynecol. Oncol. 109, 394–402 (2008). 18423823 10.1016/j.ygyno.2008.01.038 PMC2735772

[b12] Suh Y., Amelio I., Urbano T. G. & Tavassoli M. Clinical update on cancer: molecular oncology of head and neck cancer. Cell Death Dis. 5, e1018 (2014). 24457962 10.1038/cddis.2013.548 PMC4040714

[b13] Chu Q. S. . A phase I open-labeled, single-arm, dose-escalation, study of dichloroacetate (DCA) in patients with advanced solid tumors. Invest. New Drugs. 33, 603–610 (2015). 25762000 10.1007/s10637-015-0221-y

[b14] Lin G. . Chung, Dichloroacetate induces autophagy in colorectal cancer cells and tumours. Br. J. Cancer 111, 375–385 (2014). 24892448 10.1038/bjc.2014.281 PMC4102941

[b15] Stockwin L. H. . Newton, Sodium dichloroacetate selectively targets cells with defects in the mitochondrial ETC. Int. J. Cancer 127, 2510–2519 (2010). 20533281 10.1002/ijc.25499

[b16] Horiuchi N. . *In vitro* antitumor activity of mitomycin C derivative (RM-49) and new anticancer antibiotics (FK973) against lung cancer cell lines determined by tetrazolium dye (MTT) assay. Cancer Chemother. Pharmacol. 22, 246–50 (1988). 2842080 10.1007/BF00273419

[b17] Seidler E. & Wohlrab F. Suitability of monotetrazolium salt MTT derivatives for histochemical dehydrogenase demonstration. Acta Histochem. 46, 202–208 (1973). 4207940

[b18] Darzynkiewicz Z. . Features of apoptotic cells measured by flow cytometry. Cytometry 13, 795–808 (1992). 1333943 10.1002/cyto.990130802

[b19] Gorczyca W., Melamed M. R. & Darzynkiewicz Z. Apoptosis of S-phase HL-60 cells induced by DNA topoisomerase inhibitors: detection of DNA strand breaks by flow cytometry using the *in situ* nick translation assay. Toxicol. Lett. 67, 249–258 (1993). 8383887 10.1016/0378-4274(93)90060-b

[b20] Franĕk F., Vomastek T. & Dolníková J. Fragmented DNA and apoptotic bodies document the programmed way of cell death in hybridoma cultures. Cytotechnology 9, 117–123 (1992). 1369164 10.1007/BF02521738

[b21] Sen S. Programmed cell death: concept, mechanism and control. Biol. Rev. Camb. Philos. Soc. 67, 287–319 (1992). 1420728 10.1111/j.1469-185x.1992.tb00727.x

[b22] Mullen P., Ritchie A., Langdon S. P. & Miller W. R. Effect of Matrigel on the tumorigenicity of human breast and ovarian carcinoma cell lines. Int. J. Cancer 17, 67, 816–820 (1996). 8824553 10.1002/(SICI)1097-0215(19960917)67:6<816::AID-IJC10>3.0.CO;2-#

[b23] Carlsson G., Gullberg B. & Hafström L. Estimation of liver tumor volume using different formulas - an experimental study in rats. J. Cancer Res. Clin. Oncol. 105, 20–23 (1983). 6833336 10.1007/BF00391826 PMC12252809

[b24] Yang Y., Wang J., Zhang X., Lu W. & Zhang Q. A novel mixed micelle gel with thermo-sensitive property for the local delivery of docetaxel. J. Control Release 135, 175–182 (2009). 19331864 10.1016/j.jconrel.2009.01.007

[b25] Tang X. . A mechanically-induced colon cancer cell population shows increased metastatic potential. Mol. Cancer 13, 131 (2014). 24884630 10.1186/1476-4598-13-131 PMC4072622

[b26] Misra S. K. . Trimodal Therapy: Combining Hyperthermia with Repurposed Bexarotene and Ultrasound for Treating Liver Cancer. ACS Nano 10.1021/acsnano.5b05974 (2015). PMC4820022 26435333

[b27] Misra S. K., Jensen T. W. & Pan D. Enriched inhibition of cancer and stem-like cancer cells via STAT-3 modulating niclocelles. Nanoscale 7, 7127–7132 (2015). 25785368 10.1039/c5nr00403a

[b28] Malika L. . Protein kinase PKC delta and c-Abl are required for mitochondrial apoptosis induction by genotoxic stress in the absence of p53, p73 and Fas receptor. FEBS Lett. 580, 2547–2552 (2006). 16631755 10.1016/j.febslet.2006.03.089

[b29] Vibet S. . Differential subcellular distribution of mitoxantrone in relation to chemosensitization in two human breast cancer cell lines. Drug Met. Disp. 35, 822–828 (2007). 10.1124/dmd.106.013474 17296624

[b30] Lu C. W., Lin S. C., Chen K. F., Lai Y. Y. & Tsai S. J. Induction of pyruvate dehydrogenase kinase-3 by hypoxia-inducible factor-1 promotes metabolic switch and drug resistance. J. Biol. Chem. 283, 28106–28114 (2008). 18718909 10.1074/jbc.M803508200 PMC2661383

[b31] Bonnet S. . A mitochondria-K+ channel axis is suppressed in cancer and its normalization promotes apoptosis and inhibits cancer growth. Cancer Cell 11 37–51 (2007). 17222789 10.1016/j.ccr.2006.10.020

[b32] Michelakis E. D., Webster L. & Mackey J. R. Dichloroacetate (DCA) as a potential metabolic-targeting therapy for cancer. Br. J. Cancer 99, 989–994 (2008). 18766181 10.1038/sj.bjc.6604554 PMC2567082

[b33] Sun R. C. . Reversal of the glycolytic phenotype by dichloroacetate inhibits metastatic breast cancer cell growth *in vitro* and *in vivo*. Breast Cancer Res. Treat. 120, 253–260 (2010). 19543830 10.1007/s10549-009-0435-9

[b34] Zheng M. F., Shen S. Y. & Huang W. D. DCA increases the antitumor effects of capecitabine in a mouse B16 melanoma allograft and a human non-small cell lung cancer A549 xenograft. Cancer Chemother. Pharmacol. 72, 1031–1041 (2013). 24043136 10.1007/s00280-013-2281-z

[b35] Lin G. . Dichloroacetate induces autophagy in colorectal cancer cells and tumours. Br. J. Cancer 111, 375–385 (2014). 24892448 10.1038/bjc.2014.281 PMC4102941

[b36] Misra S. K., Ye M., Kim S. & Pan D. Highly efficient anti-cancer therapy using scorpion ‘NanoVenin’. Chem. Commun. 50, 13220–13223 (2014). 10.1039/c4cc04748f 25061638

[b37] Misra S. K., Ye M., Kim S. & Pan D. Defined nanoscale chemistry influences delivery of peptido-toxins for cancer therapy. PLos One 10, e0125908 (2015). 26030072 10.1371/journal.pone.0125908 PMC4452514

[b38] Nakanishi T., Morita M., Murakami H., Sagara T. & Nakashima N. Structure and electrochemistry of self-organized fullerene-lipid bilayer films. Chemistry 8, 1641–1648 (2002). 11933092 10.1002/1521-3765(20020402)8:7<1641::aid-chem1641>3.0.co;2-4

[b39] Xu C. . Self-assembled nanoparticles from hyaluronic acid-paclitaxel prodrugs for direct cytosolic delivery and enhanced antitumor activity. Int. J. Pharm. 493, 172–181 (2015). 26232702 10.1016/j.ijpharm.2015.07.069

[b40] Graf N. . α(V)β(3) integrin-targeted PLGA-PEG nanoparticles for enhanced anti-tumor efficacy of a Pt(IV) prodrug. ACS Nano 6, 4530–4539 (2012). 22584163 10.1021/nn301148e PMC3358506

