# Supplementary material for: Pro-haloacetate Nanoparticles for Efficient Cancer Therapy via Pyruvate Dehydrogenase Kinase Modulation
Source: Sci Rep. 2016 Jun 21;6:28196. doi: 10.1038/srep28196 (PMC4914936; doi:10.1038/srep28196)
Supplement: Supplementary Information [file srep28196-s1.doc]

Supporting information

**Pro-haloacetate Nanoparticles for Efficient Cancer Therapy *via* Pyruvate Dehydrogenase Kinase Modulation**

*Santosh K. Misra, Mao Ye, Fatemeh Ostadhossein and Dipanjan Pan**

Departments of Bioengineering, Materials Science and Engineering and Beckman Institute, University of Illinois at Urbana-Champaign

Mills Breast Cancer Institute, and Carle Foundation Hospital, Urbana, Illinois 61801, USA

*Corresponding author e-mail: [dipanjan@illinois.edu](mailto:dipanjan@illinois.edu)

**Figure S1.** Synthesis of haloacetate-prodrugs.

**Table S1.** Physico-chemical characterization of halo-acetate nanoparticles.

| **Nanoparticles** | **Hydrodynamic diameter (number averaged)/nm** | **Hydrodynamic diameter (intensity averaged)/nm** | **Hydrodynamic diameter (volume averaged)/nm** | **Electrophoretic potential (Zeta)/mV** | **Polydispersity** |
| --- | --- | --- | --- | --- | --- |
| Pro-DCA-NP | 39±02 | 64±02 | 48±03 | -20±05 | 0.23±0.04 |
| Pro-MCA-NP | 208±34 | 215±38 | 217±38 | -25±05 | 0.34±0.05 |
| Pro-DBA-NP | 99±17 | 101±19 | 100±18 | -22±05 | 0.85±0.08 |
| Lipid-NP | 93±10 | 64±02 | 48±03 | -35±05 | ND* |

*Not defined.

**Table S2.** Energy minimized length of various molecules involved in studies. Energy minimization was performed with MMFF94x force field with 0.1 RMS kcal/mol gradients.

| **Molecules** | **Structure** | **Distance (Å)** |
| --- | --- | --- |
| DCA | 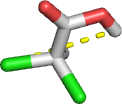 | 4.1 |
| MCA | 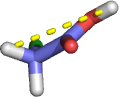 | 3.9 |
| DBA | 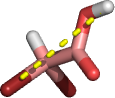 | 4.6 |
| Pro-DCA | 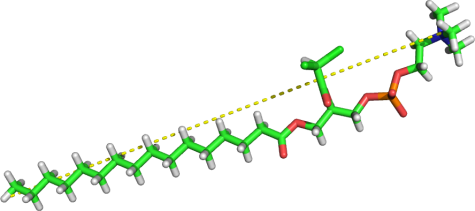 | 33.1 |
| Pro-MCA | 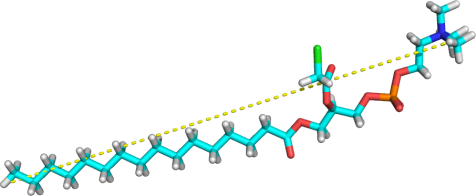 | 33.2 |
| Pro-DBA | 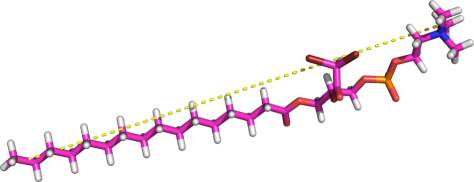 | 33.1 |
| Lyso PC | 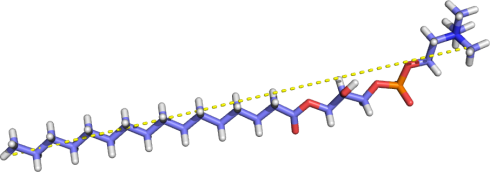 | 33.4 |


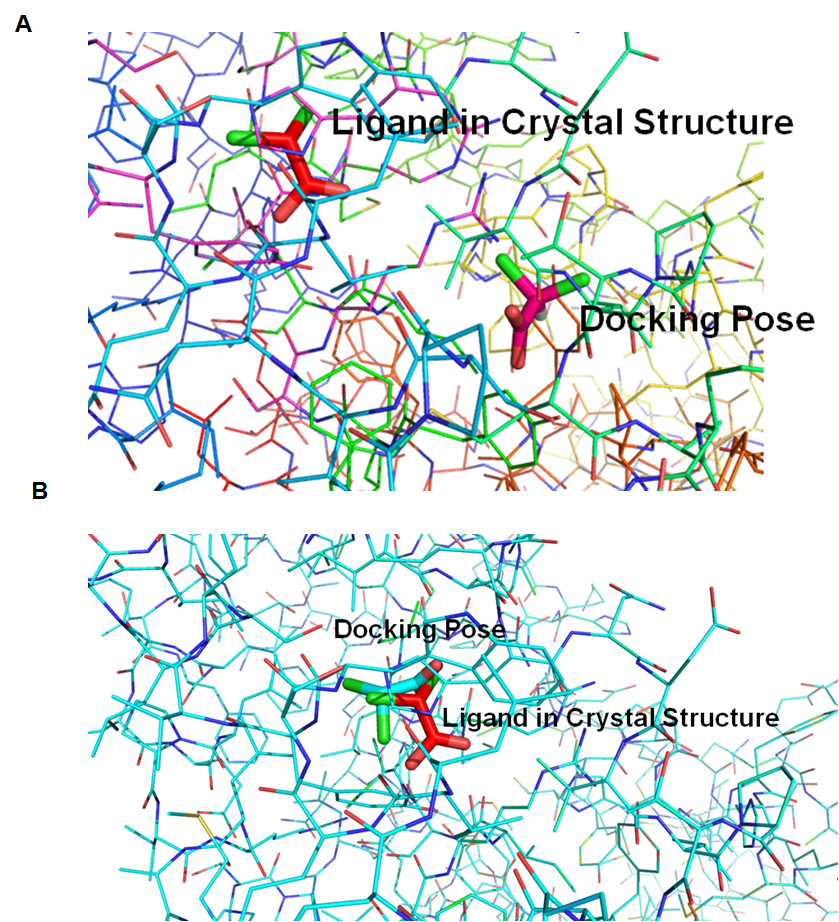


**Figure S2.** Optimization of docking poses. (a) DCA docked to the 2BU8 target (DCA removed out). Docking pose of DCA located in other site after choosing the proposed binding pocket (Composed by L53, Y80, S83, I111, R112, H115, S153, R154, I157, R158, I161) or binding pocket composed by some of these residues; (b) ILE 111 site as the binding site for DCA docking pose and located at same binding pocket with original ligand.

**Pro-DCA-NP**

**BT549**

**Pro-MCA-NP**

**Pro-DBA-NP**

**DCA**

**MCA**

**DBA**

**Figure S3.** Representative bright field images for BT549 cells, untreated or treated with different formulations at 100 µM concentration of haloacetates in free or form of Pro-drug-NP acquired after 48h of treatment.
